# The H3K9 methyltransferase Setdb1 regulates TLR4-mediated inflammatory responses in macrophages

**DOI:** 10.1038/srep28845

**Published:** 2016-06-28

**Authors:** Rumi Hachiya, Takuya Shiihashi, Ibuki Shirakawa, Yorihiro Iwasaki, Yoshihiro Matsumura, Yumiko Oishi, Yukiteru Nakayama, Yoshihiro Miyamoto, Ichiro Manabe, Kozue Ochi, Miyako Tanaka, Nobuhito Goda, Juro Sakai, Takayoshi Suganami, Yoshihiro Ogawa

**Affiliations:** 1 Department of Molecular Endocrinology and Metabolism, Graduate School of Medical and Dental Sciences, Tokyo Medical and Dental University, 1-5-45 Yushima, Bunkyo-ku, Tokyo, 113-8510, Japan; 2 Department of Life Science and Medical Bio-Science, School of Advanced Science and Engineering, Waseda University, 2-2 Wakamatsu-cho, Shinjuku-ku,Tokyo, 162-8480, Japan; 3 Department of Organ Network and Metabolism, Graduate School of Medical and Dental Sciences, Tokyo Medical and Dental University, 1-5-45 Yushima, Bunkyo-ku, Tokyo, 113-8510, Japan; 4 Division of Metabolic Medicine, Research Center for Advanced Science and Technology, University of Tokyo, 4-6-1 Komaba, Meguro-ku, Tokyo, 153-8904, Japan; 5 Department of Cellular and Molecular Medicine, Medical Research Institute, Tokyo Medical and Dental University, 1-5-45 Yushima, Bunkyo-ku, Tokyo, 113-8510, Japan; 6 Department of Cardiovascular Medicine, Graduate School of Medicine and Faculty of Medicine, University of Tokyo, 7-3-1 Hongo, Bunkyo-ku, Tokyo, 113-8655, Japan; 7 Department of Preventive Cardiology, National Cerebral and Cardiovascular Center, 5-7-1 Fujishiro-dai, Suita, Osaka, 565-0873, Japan; 8 Department of Aging Research, Graduate School of Medicine, Chiba University, 1-8-1 Inohana, Chuo-ku, Chiba-shi, Chiba, 260-8670, Japan; 9 Department of Molecular Medicine and Metabolism, Research Institute of Environmental Medicine, Nagoya University, Furo-cho, Chikusa-ku, Nagoya, 464-8601, Japan; 10 Japan Science and Technology Agency, PRESTO, 7 Goban-cho, Chiyoda-ku, Tokyo, 102-0076, Japan; 11 Japan Agency for Medical Research and Development, AMED-CREST, 1-7-1 Otemachi, Chiyoda-ku, Tokyo, 100-0004, Japan; 12 Research Institute of Environmental Medicine, Nagoya University, Furo-cho, Chikusa-ku, Nagoya, 464-8601, Japan

## Abstract

Proinflammatory cytokine production in macrophages involves multiple regulatory mechanisms, which are affected by environmental and intrinsic stress. In particular, accumulating evidence has suggested epigenetic control of macrophage differentiation and function mainly *in vitro*. SET domain, bifurcated 1 (Setdb1, also known as Eset) is a histone 3 lysine 9 (H3K9)-specific methyltransferase and is essential for early development of embryos. Here we demonstrate that Setdb1 in macrophages potently suppresses Toll-like receptor 4 (TLR4)-mediated expression of proinflammatory cytokines including interleukin-6 through its methyltransferase activity. As a molecular mechanism, Setdb1-deficiency decreases the basal H3K9 methylation levels and augments TLR4-mediated NF-κB recruitment on the proximal promoter region of interleukin-6, thereby accelerating interleukin-6 promoter activity. Moreover, macrophage-specific Setdb1-knockout mice exhibit higher serum interleukin-6 concentrations in response to lipopolysaccharide challenge and are more susceptible to endotoxin shock than wildtype mice. This study provides evidence that the H3K9 methyltransferase Setdb1 is a novel epigenetic regulator of proinflammatory cytokine expression in macrophages *in vitro* and *in vivo*. Our data will shed insight into the better understanding of how the immune system reacts to a variety of conditions.

One of the key features of macrophages is to produce cytokines and chemokines in response to a variety of environmental and intrinsic stress. Since sustained and/or excessive production of proinflammatory cytokines leads to chronic inflammation, the process is tightly regulated by multiple feedback mechanisms. Among a number of proinflammatory signals, Toll-like receptor 4 (TLR4), a major pathogen sensor for the recognition of lipopolysaccharide (LPS), has been most intensively investigated. Indeed, there is considerable evidence about transcriptional, translational, and post-translational regulation of TLR4-mediated proinflammatory cytokine production^1^,^2^,^3^. Recently, we have reported that members of the activating transcription factor (ATF)/cAMP response element binding protein (CREB) family of transcription factors are involved in a positive (e.g., ATF4) or a negative (e.g., ATF3) regulation of TLR4 signaling^4^,^5^. In addition to transcription factors, epigenetic mechanisms, particularly histone modifications, modulate inflammatory responses positively or negatively depending on the modification types^6^. For example, methylation of histone 3 lysine 4 (H3K4) is associated with activation of transcription, whereas that of H3K9 and H3K27 is related to transcriptional repression^7^.

It is known that histone modifications are affected by intracellular and extracellular conditions. In this regard, Villeneuve *et al*. reported that high glucose conditions may decrease H3K9 methylation levels, thereby accelerating proinflammatory cytokine expression in vascular smooth muscle cells^8^. Although H3K9 methylation was once regarded as stable or irreversible, accumulating evidence has revealed that dynamic changes in H3K9 methylation occur at proinflammatory genes upon stimulation^8^,^9^,^10^. Nevertheless, the molecular mechanism of how H3K9 methylation is regulated on the promoter of proinflammatory cytokines is not fully understood.

SET domain, bifurcated 1 (Setdb1; also known as Eset) is an H3K9 methyltransferase and represses gene expression through trimethylation of H3K9 (H3K9me3). Setdb1 is essential for early development of embryo and global Setdb1-knockout mice are embryonic lethal^11^. On the other hand, gene amplification of Setdb1 is observed in several cancer cell types such as lung cancer^12^, melanoma^13^, and hepatocellular carcinoma^14^, suggesting the role of Setdb1 in carcinogenesis. Moreover, Setdb1 plays a role in differentiation of adipocytes^15^,^16^ and chondrocytes^17^. However, the role of Setdb1 in immune cells or inflammation remains to be elucidated.

Here we demonstrate that Setdb1-deficiency in macrophages increases TLR4-mediated expression of proinflammatory cytokines including interleukin-6 (IL6). In line with this, macrophage-specific Setdb1-knockout mice are more susceptible to LPS-induced endotoxin shock than wildtype mice. As a molecular mechanism, Setdb1-deficiency decreases the basal H3K9 methylation levels on the proximal promoter region of IL6 and augments the LPS-induced NF-κB p65 recruitment to the site, thereby accelerating IL6 promoter activity. This study provides evidence that Setdb1 is a novel epigenetic regulator of proinflammatory cytokine expression in macrophages.

## Results

### Setdb1 suppresses TLR4-mediated proinflammatory cytokine expression

**Figure 1:**
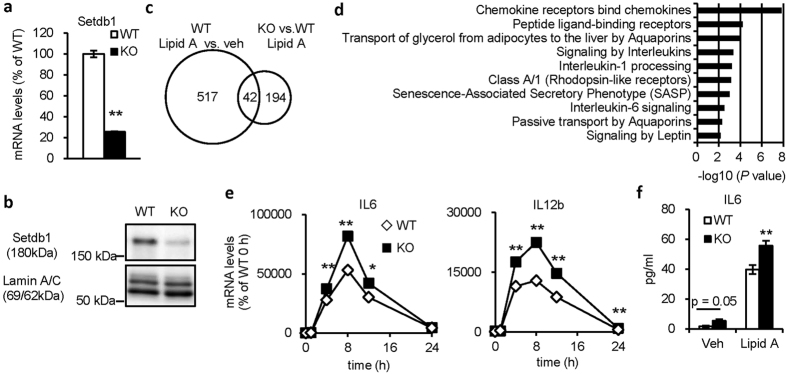
Setdb1 suppresses TLR4-mediated proinflammatory cytokine expression. (**a**,**b**) Expression of Setdb1 in peritoneal macrophages from LysM Cre/+:Setdb1 fl/fl (KO) and +/+:Setdb1 fl/fl (WT) mice. (**a**) mRNA expression (**P < 0.01, n = 3) and (**b**) representative Western blots of nuclear extracts. (**c**) Venn diagram of 42 genes upregulated by lipid A treatment in KO macrophages. KO and WT peritoneal macrophages were treated with lipid A (10 ng/ml) or vehicle for 4 hours. (**d**) Gene ontology analysis of the 42 genes. The top 10 significantly enriched categories are shown. (**e**) Time course of mRNA expression of proinflammatory cytokines in KO and WT peritoneal macrophages (*P < 0.05, **P < 0.01 vs. WT, n = 3). Cells were treated with lipid A (10 ng/ml) or vehicle for indicated times. (**f**) IL6 levels in the culture media (**P < 0.01 vs. WT, n = 3). Cells were treated with lipid A (10 ng/ml) or vehicle for 8 hours.

Since global Setdb1-knockout mice are embryonic lethal^11^, we generated macrophage-specific Setdb1-knockout mice by crossing Setdb1 fl/fl mice with LysM Cre/+ mice. In this study, +/+:Setdb1 fl/fl mice (designated WT mice) were used as a control group and LysM Cre/+:Setdb1 fl/fl mice (designated KO mice) were used as an experimental group. We examined mRNA and protein expression of Setdb1 in peritoneal macrophages and confirmed the knockout efficiency (Fig. 1a,b). There was no difference in the number of thioglycollate-elicited peritoneal macrophages defined with flow cytometry between the genotypes (Supplementary Fig. S1).

In an attempt to elucidate the role of Setdb1 in the TLR4-mediated inflammatory responses, we performed cDNA microarray analysis using lipid A (the active component of LPS)-stimulated peritoneal macrophages from KO and WT mice. A total of 559 genes were upregulated (>2-fold) in WT macrophages by lipid A treatment, among which 42 genes were further increased (>3-fold) in KO macrophages (Fig. 1c). We focused on these 42 genes, in which Gene ontology analysis using the Reactome database revealed that several signaling pathways associated with interleukins and chemokines were significantly enriched (Fig. 1d).

We confirmed that the lipid A-induced mRNA expression of IL6 and IL12b were augmented in peritoneal macrophages from KO mice relative to WT mice (Fig. 1e). Consistently, peritoneal macrophages from KO mice showed increased secretion of IL6 by lipid A stimulation in the media relative to those from WT mice (Fig. 1f). Meanwhile, the basal levels of IL6 in vehicle-treated KO macrophages tended to be high relative to those in vehicle-treated WT, which did not reach the statistical significance (Fig. 1e,f). In addition, Setdb1 is dispensable for mRNA expression of the genes related to the TLR4-proximal signaling pathways (Supplementary Fig. S2). These observations suggest that Setdb1 suppresses TLR4-mediated proinflammatory cytokine expression.

### Setdb1 increases H3K9 methylation levels and suppresses transcriptional activity of IL6 promoter

**Figure 2:**
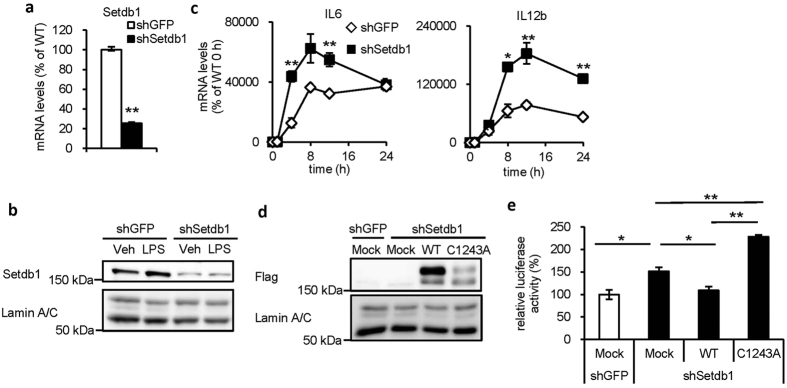
Setdb1 suppresses IL6 transcription by H3K9 methyltransferase activity. (**a**,**b**) Retrovirus-mediated knockdown of Setdb1 in J774.1 macrophage cell line. Cells infected with the control shRNA are indicated as shGFP. (**a**) mRNA expression (**P < 0.01, n = 3) and (**b**) representative Western blots of nuclear extracts. (**c**) Effect of Setdb1 knockdown on mRNA expression of proinflammatory cytokines in J774.1 macrophages. Cells were treated with LPS (100 ng/ml) or vehicle for indicated times (*P < 0.05, **P < 0.01 vs. shGFP, n = 3). (**d**,**e**) Role of the lysine methyltransferase activity in Setdb1-mediated IL6 transcription. shGFP- and shSetdb1-J774.1 macrophages transiently transfected with empty vector (Mock), wildtype Setdb1 (WT) or lysine methyltransferase-defective Setdb1 (C1243A) were used. (**d**) Representative Western blots of nuclear extracts. (**e**) A luciferase assay using the proximal IL6 promoter (from -581 bp) (*P < 0.05, **P < 0.01, n = 3).

To understand the molecular mechanism of how Setdb1 regulates proinflammatory cytokine expression, we used J774.1 macrophage cell line. Retrovirus-mediated knockdown of Setdb1 was confirmed by real-time PCR and Western blotting (Fig. 2a,b). Of note, there was no appreciable change in Setdb1 protein levels between vehicle- and LPS-treated J774.1 macrophages (Fig. 2b). Similar to peritoneal macrophages, Setdb1 knockdown significantly augmented the lipid A-induced mRNA expression of IL6 and IL12b in J774.1 macrophages (Fig. 2c).

To examine the role of Setdb1 in IL6 transcription, Setdb1-knockdown J774.1 macrophages were transiently transfected with Flag-tagged wildtype (WT) Setdb1 or lysine methyltransferase-defective (C1243A) Setdb1 (Fig. 2d) and were subjected to a luciferase assay with the proximal IL6 promoter construct (from−581 bp) (Fig. 2e). Setdb1 knockdown markedly increased the LPS-induced luciferase activity (Fig. 2e, shGFP Mock vs. shSetdb1 Mock), which was effectively suppressed by replenishment of wildtype Setdb1 (Fig. 2e, shSetdb1 Mock vs. shSetdb1 WT). In contrast, transfection of C1243A Setdb1 did not show the suppressive effect and rather increased the activity (Fig. 2e, shSetdb1 Mock vs. shSetdb1 C1243A). In this study, the expression levels of the mutant Setdb1 were apparently lower than those of WT Setdb1 (Fig. 2d), which may raise the possibility that the lysine methyltransferase activity affects intracellular localization and/or protein stability of Setdb1. Indeed, recent evidence has suggested the involvement of multiple mechanisms including nuclear transport and proteasome degradation in nuclear Setdb1 accumulation^18^,^19^.

**Figure 3:**
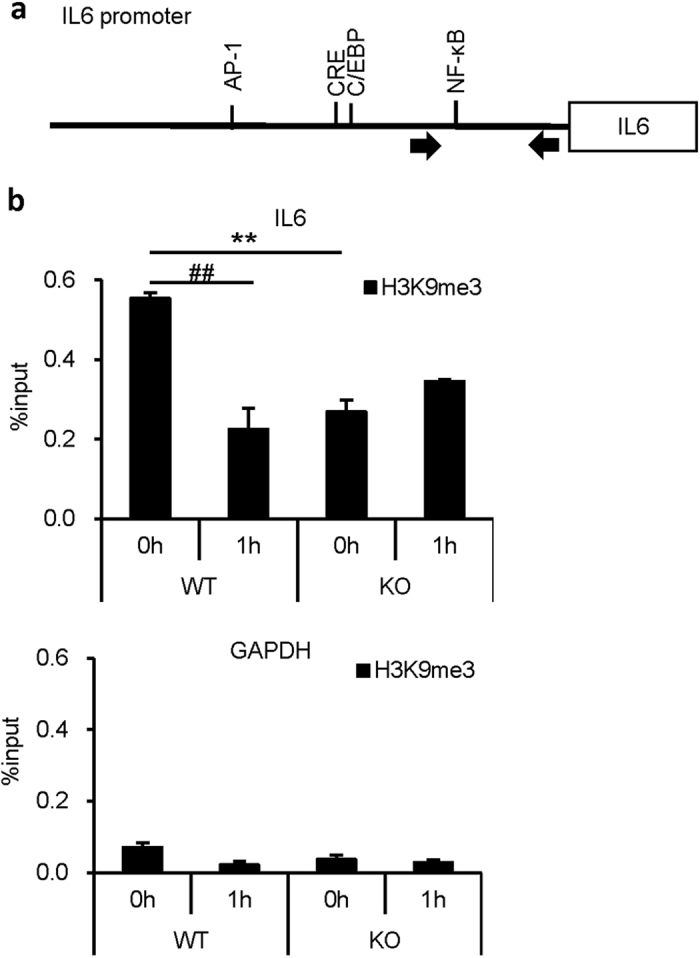
Setdb1-mediated H3K9 methylation on the IL6 promoter. (**a**) Structure of the proximal IL6 promoter. The region containing the NF-κB-binding site was amplified for ChIP-qPCR analysis. Arrows indicate the qPCR primers. (**b**) A ChIP assay of H3K9me3 in peritoneal macrophages from KO and WT mice. Cells were treated with lipid A (100 ng/ml) or vehicle for indicated times. The results were presented as percent input. GAPDH gene was used as a negative control. (**P < 0.01 vs. WT, ^##^P < 0.01 vs. 0 h, n = 3).

We next performed a chromatin immunoprecipitation (ChIP) assay using anti-H3K9me3 antibody to examine the effect of Setdb1 catalytic activity on the IL6 promoter (Fig. 3a,b). The basal levels of H3K9me3 were significantly low in KO macrophages relative to WT macrophages. Consistent with the previous reports^9^,^10^, the H3K9me3 levels were markedly reduced at 1 h after LPS treatment and returned to the baseline levels at 24 h (Fig. 3b and data not shown). On the other hand, the levels were stable in KO macrophages (Fig. 3b). These observations taken together indicate that Setdb1 increases H3K9 trimethylation on the IL6 promoter and suppresses IL6 transcription by its H3K9 methyltransferase activity.

### Setdb1-deficiency increases NF-κB p65 recruitment to the IL6 promoter

**Figure 4:**
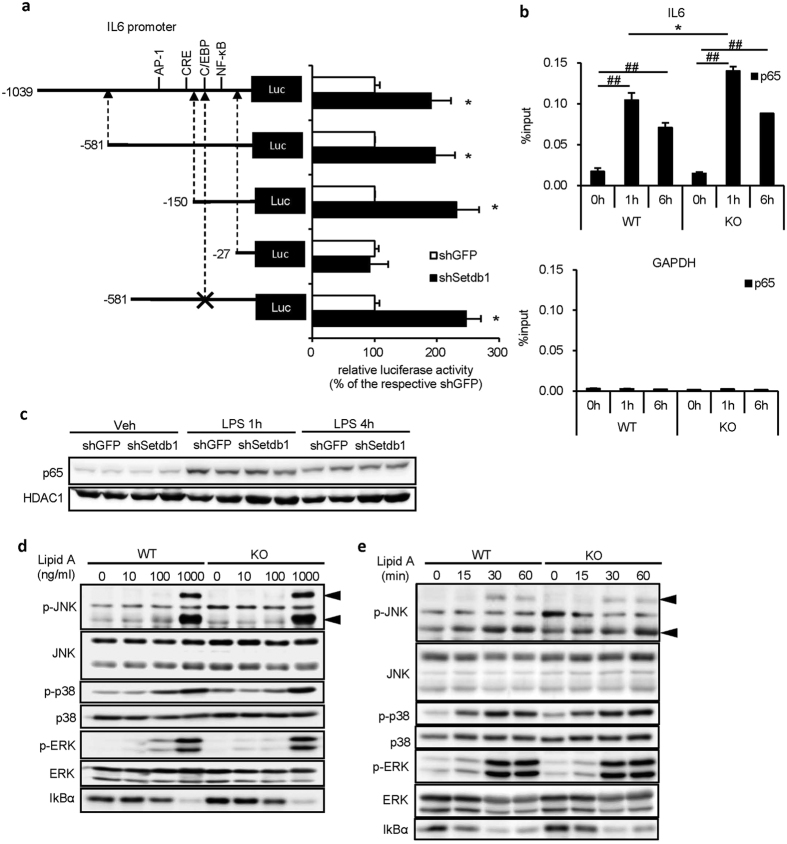
Setdb1-deficiency increases NF-κB p65 recruitment to the IL6 promoter. (**a**) An IL6 promoter luciferase assay in J774.1 macrophages infected with shSetdb1 or shGFP retrovirus. Cells were transiently transfected with a series of truncated or mutated (indicated with “x”) IL6 promoters and treated with LPS (100 ng/ml). (*P < 0.05, n = 3). (**b**) A ChIP assay of p65 in peritoneal macrophages from KO and WT mice. Cells were treated with lipid A (100 ng/ml) or vehicle for indicated times. GAPDH gene was used as a negative control. (*P < 0.05 vs. WT, ^##^P < 0.01 vs. 0 h, n = 3). (**c**) Representative Western blots for p65 in nuclear extracts from shSetdb1- and shGFP-J774.1 macrophages. Cells were treated with LPS (100 ng/ml) or vehicle for indicated times. (**d**,**e**) Representative Western blots for phosphorylated JNK, p38, and ERK and IκBα. The whole cell lysates were used. Peritoneal macrophages from KO and WT mice were treated (**d**) with the indicated concentrations of lipid A or vehicle for 15 minutes or (**e**) with 100 ng/ml of lipid A or vehicle for indicated times.

To clarify the molecular mechanism of how Setdb1 regulates IL6 transcription, we performed a luciferase assay with a series of truncated IL6 promoter constructs and found that the IL6 proximal promoter region (–150 bp to –27 bp) containing the C/EBP- and NF-κB-binding sites was indispensable for the Setdb1-mediated transcriptional repression (Fig. 4a). Since mutant sequences in the C/EBP-binding site did not affect the IL6 promoter activity (Fig. 4a), we conducted a ChIP assay using anti-NF-κB p65 (p65) antibody (Fig. 4b). In WT peritoneal macrophages, lipid A treatment increased p65 recruitment to the IL6 promoter as previously reported^20^, which was augmented in KO peritoneal macrophages. On the other hand, Western blotting for nuclear localization of NF-κB p65, phosphorylation of mitogen-activated protein kinases and degradation of inhibitor of nuclear factor κBα (IκBα) failed to detect appreciable difference in the TLR4-mediated intracellular signaling pathways in KO and WT peritoneal macrophages (Fig. 4c,d). These observations suggest that Setdb1-deficiency increases NF-κB p65 recruitment to the IL6 promoter.

### Setdb1-deficient mice are susceptible to LPS challenge

**Figure 5:**
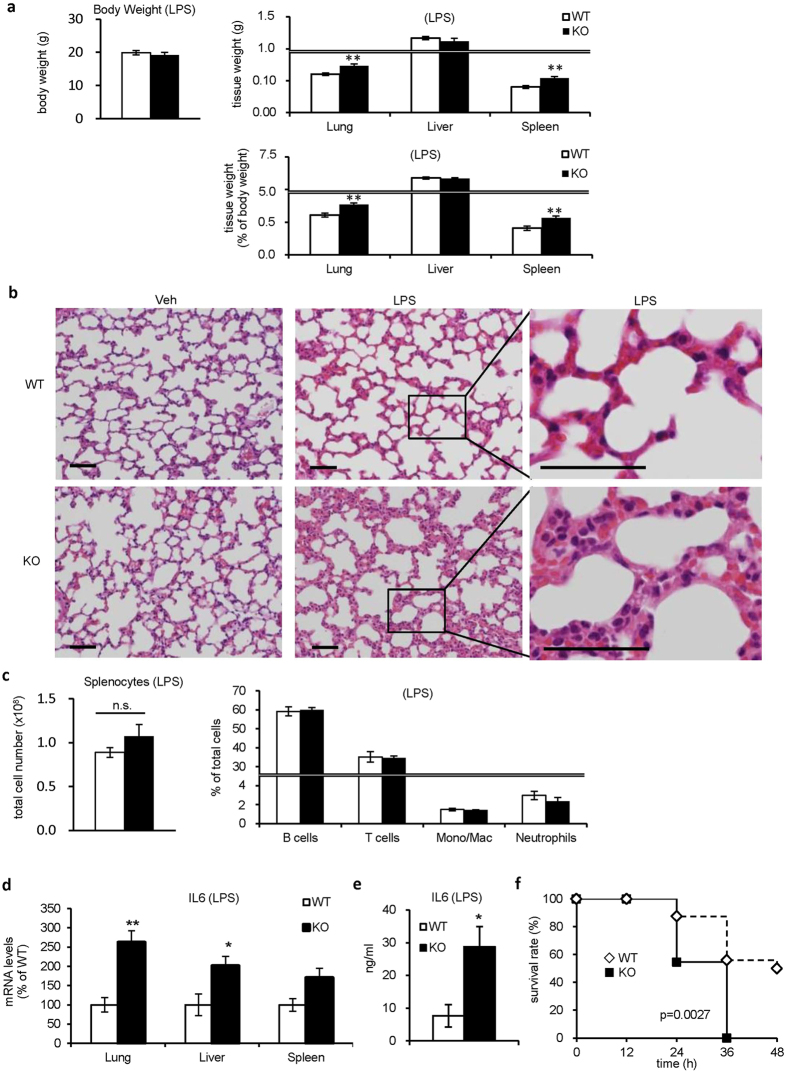
Setdb1-deficient mice are susceptible to LPS challenge. (**a**–**d**) LPS (1 mg/kg body weight) was injected intraperitoneally to KO and WT mice and euthanized at 24 hours after LPS challenge. (*P < 0.05, **P < 0.01, n = 7) (**a**) Body weight and tissue weights. (**b**) Hematoxylin and eosin staining of the lungs. Scale bars, 50 μm. (**c**) The total cell number and population of splenocytes were analyzed with flow cytometry. B lymphocytes, B220^+^; T lymphocytes, CD3ε^+^; monocytes/macrophages, CD11b^+^Ly6G^−^; neutrophils, CD11b^+^Ly6G^+^. (**d**) mRNA expression of IL6 in various tissues. (**e**) Serum IL6 concentrations at 8 hours. (**f**) Survival curve of KO and WT mice after the lethal dose of LPS injection (15 mg/kg body weight) (WT, n = 16; KO, n = 11).

To elucidate the pathophysiological significance of Setdb1 *in vivo*, we injected the sublethal dose of LPS (1 mg/kg body weight) intraperitoneally to KO and WT mice. Without LPS injection, there was only a slight increase in the spleen weight in KO mice relative to WT mice without LPS injection (Supplementary Fig. S3a). The weight of the lungs and spleen were significantly increased in KO mice relative to WT mice 24 hours after the LPS injection (Fig. 5a). Histological examination of the lungs revealed more severe inflammatory changes such as marked mononuclear cell infiltration, interalveolar septal thickening and interstitial edema in KO mice than in WT mice (Fig. 5b). In the spleen, there was no significant difference in the number and population of splenocytes between KO and WT mice with or without LPS injection (Fig. 5c and Supplementary Fig. S3b). In line with our *in vitro* data, the LPS-induced increase in IL6 mRNA expression in various tissues and serum IL6 concentrations was augmented in KO mice relative to WT mice (Fig. 5d,e). Furthermore, we injected the lethal dose of LPS (15 mg/kg body weight) intraperitoneally to KO and WT mice and found that KO mice were highly susceptible to the LPS-induced endotoxin shock relative to WT mice (Fig. 5f). Of note, analysis using flow cytometry and quantitative real-time PCR showed no appreciable difference in macrophage polarization in various tissues (Supplementary Figs S1b and S3c). Collectively, these observations suggest that Setdb1 suppresses TLR4-mediated inflammatory responses *in vivo*.

## Discussion

The innate immune system should maintain a balance between effective pathogen defense and appropriate termination of inflammation. Indeed, proinflammatory cytokine production involves multiple regulatory mechanisms, which are affected by environmental and intrinsic stress. In particular, accumulating evidence has suggested epigenetic control of myeloid cell differentiation and function *in vitro*^6^, whereas its role *in vivo* remains to be fully elucidated. In this study, we demonstrated that macrophage-specific Setdb1 KO mice show increased susceptibility to the LPS-induced septic shock. In line with this, Setdb1 negatively regulates TLR4-mediated proinflammatory cytokine expression in cultured macrophages. To examine the molecular mechanism, we focused on IL6 since there was a rapid and evident change in the H3K9me3 levels on the proximal region of IL6 promoter. Then, we found that Setdb1 is required for H3K9 methylation, thereby regulating IL6 transcription through its methyltransferase activity. So far, Setdb1 has been linked to early development of embryos, differentiation of adipocytes^15^,^16^ and chondrocytes^17^, and carcinogenesis^12^,^13^,^14^. To the best of our knowledge, this is the first report to show that Setdb1 is a novel epigenetic regulator of proinflammatory cytokine production in immune cells. Based on our observations and the recent evidence for the role of epigenetic regulation in alternatively activated macrophage phenotype^21^, it is conceivable that histone modifications contribute to fine-tuning of the innate immune system *in vivo*.

H3K9 methylation is a dynamic process. Indeed, LPS stimulation induces a marked reduction of the H3K9me3 levels in macrophages. In this study, Setdb1 deficiency markedly reduces the basal levels of H3K9 methylation without affecting those of IL6 expression, which is followed by increased NF-κB recruitment upon LPS stimulation. These observations led us to speculate that the resting-state H3K9 methylation levels play a role as a gatekeeper that regulates NF-κB accessibility to the IL6 promoter. Interestingly, Aof1, an H3K9 demethylase, is recruited to the promoter region of proinflammatory cytokines such as IL12b and CCL22 by LPS stimulation in dendritic cells, thereby removing H3K9 methylation and increasing NF-κB p65 recruitment to induce their transcription^10^. Since there was no further TLR4-mediated reduction in H3K9me3 in Setdb1 KO macrophages, it is conceivable that Setdb1 is functionally linked to Aof1. Accordingly, it is interesting to know how chromatin remodeling occurs and how it affects proinflammatory cytokine production in acute TLR4-mediated cellular responses.

It is important to address how Setdb1 regulates the H3K9me3 levels on the IL6 promoter. In this regard, Yoshida *et al*. reported a direct binding of ATF7 on the CXCL2 promoter, where ATF7 recruits G9a, another H3K9 methyltransferase, thereby increasing the H3K9 methylation levels and suppressing the CXCL2 transcriptional activity^22^. Similarly, Setdb1 may also have a binding partner which regulates its histone methyltransferase activity and its recruitment to the IL6 promoter. In addition to the histone methyltransferase activity, a recent study pointed to the role of Setdb1 as a non-histone lysine methyltransferase. In hepatocellular carcinoma, amplified Setdb1 dimethylates and stabilizes mutated p53 at Lys370, which leads to cancer cell growth^14^. Currently, we do not exclude the possibility that Setdb1 exerts its repressive effect on IL6 transcription through methylation of non-histone proteins, since it is unclear whether Setdb1 is recruited to the NF-κB binding site of IL6 promoter. Nevertheless, our data suggest that Setdb1 suppresses IL6 expression through its methylation activity on H3K9.

It should be addressed how other H3K9 methyltransferases (such as Suv39h1, Suv39h2, G9a, Glp, and Setdb2 in mammals) are related to Setdb1-mediated regulation of IL6 transcription. Although there may be a compensation mechanism for Setdb1 deficiency, our preliminary data show that Setdb1 deficiency does not affect mRNA expression of other H3K9 methyltransferases in macrophages (unpublished data, Hachiya *et al*., 2016). Of note, Suv39h1, G9a, GLP, and Setdb1 participate in a multimeric complex, where their protein stability and methyltransferase activity are interdependent^23^. Therefore, Setdb1 may collaborate with other H3K9 methyltransferases to regulate IL6 expression. On the other hand, H3K9 methyltransferases in immune cells are supposed to have distinct roles depending on context such as cell types, metabolic status and their target genes^6^. For instance, Setdb2, the family member most closely related to Setdb1, mediates virus-induced susceptibility to secondary bacterial infection by regulating CXCL1 transcription^24^. In vascular smooth muscle cells, Suv39h1 methylates H3K9 on the IL6 promoter to repress IL6 expression^8^. Intriguingly, expression of Setdb2 and Suv39h1 is positively and negatively controlled by interferon-β and high glucose conditions, respectively^8^,^24^. In this regard, H3K9 methylation may modulate inflammatory response depending on a variety of cellular stress. Accordingly, it is important to know how expression and activity of H3K9 methyltransferases are regulated in immune cells, which would be helpful to understand a novel mechanism of cellular response to infection and injury.

**Figure 6:**
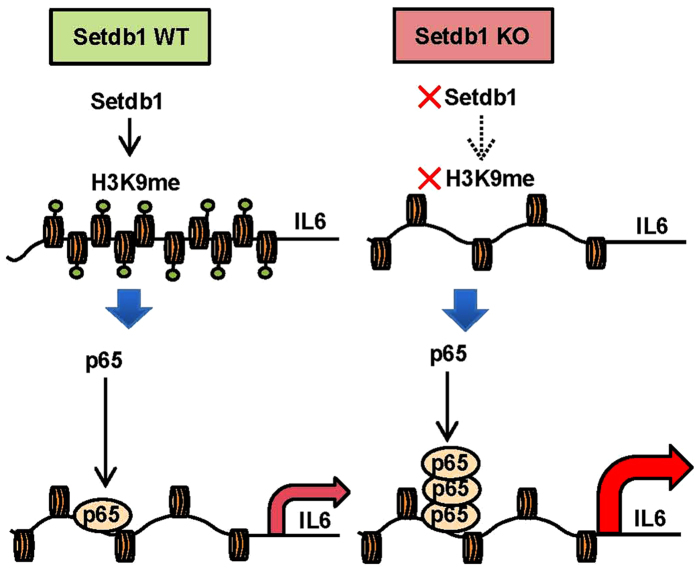
Schematic representation of the role of Setdb1 in the regulation of TLR4-mediated IL6 expression. At resting-state, Setdb1 maintains the H3K9 methylation levels of the proximal region of IL6 promoter. Upon LPS stimulation, there is a reduction of H3K9 methylation levels, which is linked to the recruitment of NF-κB p65 to the site, thereby activating IL6 transcription. The Setdb1-mediated resting-state H3K9 methylation may act as a gatekeeper that regulates NF-κB accessibility.

On the basis of our observations, we hypothesize a role of Setdb1 in TLR4-mediated proinflammatory cytokine expression as follows (Fig. 6). At resting-state, Setdb1 maintains the H3K9 methylation levels of the proximal region of IL6 promoter. Upon LPS stimulation, there is a reduction of H3K9 methylation levels, which is linked to the recruitment of NF-κB p65 to the site, thereby activating IL6 transcription. Our data suggest that the Setdb1-mediated resting-state H3K9 methylation acts as a gatekeeper that regulates NF-κB accessibility. Moreover, Setdb1 in macrophages plays an important regulatory role in the LPS-induced septic shock *in vivo*. For future directions, it is important to know how Setdb1 expression and activation is regulated in response to internal and external stimuli. In summary, we demonstrate that the H3K9 methyltransferase Setdb1 in macrophages tunes TLR4-mediated inflammatory response *in vitro* and *in vivo*. Our data will shed insight into the better understanding of how the immune system reacts to infection and injury under a variety of conditions.

## Methods

### Animals

Setdb1 fl/fl mice were kindly provided by Dr. Yoichi Shinkai (RIKEN, Saitama, Japan)^25^ and LysM Cre/+ transgenic mice were described previously^26^. Macrophage-specific Setdb1 KO mice on the C57BL/6 genetic background were produced by crossing Setdb1 fl/fl mice with LysM Cre/+ mice in a temperature-, humidity-, and light-controlled room (12 h light/dark cycles), allowed free access to water and standard chow (CE-2; CLEA Japan, Tokyo, Japan). All animal experiments were performed in accordance with the guidelines of Tokyo Medical and Dental University and approved by the Institutional Animal Care and Use Committee of Tokyo Medical and Dental University (No. 2015-008C4, No. 0160381A).

### Cells, reagents, and antibodies

Murine peritoneal macrophages were elicited by peritoneal injection of 3% sterile thioglycollate medium (3 ml per mouse; BD Biosciences, San Jose, CA). Four days after injection, peritoneal exudate cells were collected by peritoneal washing. After red blood cells lysis, the exudate cells were filtered through a 100-μm nylon net filter (Merck Millipore, Darmstadt, Germany). After 4 hours of culture, the attached cells were used as peritoneal macrophages. The J774.1 macrophage cell line was purchased from RIKEN BioResource Center. (Tsukuba, Japan). These macrophages were cultured in Dulbecco’s modified Eagle’s medium (Nacalai Tesque, Kyoto, Japan) containing 10% fetal bovine serum. LPS (L4391 for *in vitro* experiments, L2630 for *in vivo* experiments) and lipid A (L5399) were purchased from Sigma-Aldrich (St. Louis, MO). Monoclonal anti-mouse Setdb1 antibody was prepared as reported previously^16^. Antibodies used in Western blotting and chromatin immunoprecipitation assay are listed in Supplementary Table S1. Antibodies used in flow cytometric analyses were listed in Supplementary Table S2.

### cDNA microarray analysis

cDNA Microarray analysis was performed using Affymetrix GeneChip Mouse Genome 430 2.0 Arrays as described previously^27^. Genes with an average fold change >2.0 or >3.0 together with a label of “present (P)” and “increased (I)” were considered to be differentially upregulated. Pathway and gene ontology analyses were performed using the Reactome database (http://www.reactome.org/)^4^. The raw data are available on the web site of the Gene Expression Omnibus at the National Center for Biotechnology Information (GEO accession: GSE78153).

### Quantitative real-time PCR

Quantitative real-time PCR was performed as described^4^. In brief, 10 ng of cDNA was used for real-time PCR amplification with SYBR GREEN detection protocol in a thermal cycler (StepOne Plus; ThermoFisher Scientific, Waltham, MA). Primers used in this study were described in Supplementary Table S3. Data were normalized to the 36B4 levels, and analyzed using the comparative threshold cycle method.

### Plasmid construction, transfection and knockdown experiments

An expression vector encoding Flag-tagged murine Setdb1 or Setdb1 (C1243A) was a generous gift from Dr. Shinkai^25^. Knockdown-resistant forms of these vectors were generated by the inverse PCR-based site-directed mutagenesis. The rat IL6 promoter luciferase reporter plasmid and the truncated constructs were kindly provided by Dr. Toshihiro Ichiki (Kyushu University, Fukuoka, Japan)^28^. All transient transfection experiments were performed using X-tremeGENE HP DNA Transfection Reagent (Roche Diagnostic, Penzberg, Germany) according to the manufacturer’s protocol. Retrovirus-mediated knockdown of Setdb1 was performed as previously described^5^. The target sequences for shSetdb1 were 5′-GCTACATCATTGATGCCAAACTTGA-3′ as previously described^15^ and shGFP was used as a negative control.

### IL6 promoter luciferase assay

IL6 promoter luciferase assay was performed using J774.1 macrophages as described^4^ with minor modifications. In brief, 6 hours after transfection of luciferase reporter plasmids, cells were treated with LPS (100 ng/ml) for 24 hours. The luciferase activity was measured with a dual-luciferase reporter assay system (Promega, Madison, WI). All data were normalized for transfection efficiency by the division of firefly luciferase activity by renilla luciferase activity.

### Western Blotting

Whole cell lysates and nuclear fractions were prepared as described^4^. Samples were separated by SDS-PAGE and immunoblots were developed with horseradish peroxidase-conjugated secondary antibodies and a chemiluminescence kit (ECL prime; GE Healthcare, Buckinghamshire, UK) and then observed with ImageQuant LAS 4000 mini (Fujifilm, Tokyo, Japan). Full images of Western blots are shown in Supplementary Fig. S4.

### Chromatin immunoprecipitation assay

ChIP was performed as described^29^ with minor modifications. For H3K9me3 ChIP, cells were crosslinked in phosphate-buffered saline (PBS) containing 1% formaldehyde (28908, ThermoFisher Scientific) for 5 min. For p65 ChIP, cells were first crosslinked in PBS containing 2 mM ethylene glycol-bis (E3257, Sigma-Aldrich) for 30 min, and then in PBS containing 1% formaldehyde for another 10 min. Chromatin was sheared by Sonifier SLPe40 (Branson Ultrasonics, Danbury, CT) and the average DNA size (approximate 400 bp) was confirmed by Agilent 2100 Bioanalyzer (Agilent Technologies, Palo Alto, CA). Sonicated nuclear extracts were immunoprecipitated with primary antibodies listed in Supplementary Table S1. The purified DNA was quantified with quantitative real-time PCR using the primer sequences listed in Supplementary Table S4. The results are presented as a percent input value.

### ELISA

IL6 concentrations in culture media and serum were measured using Mouse IL6 Quantikine ELISA Kit (R&D Systems, Minneapolis, MN) as described previously^4^.

### Flow cytometric analyses of splenocytes and peritoneal exudate cells

Harvested spleens were suspended in phosphate-buffered saline (PBS), followed by passage through a 100-μm nylon cell strainer (BD Falcon, Bedford, MA). After red blood cells lysis, the residual cells were filtered through a 100-μm nylon net filter. Prepared splenocytes and peritoneal exudate cells were counted using Countess automated cell counter (ThermoFisher Scientific, Waltham, MA). Flow cytometric analyses for splenocytes and peritoneal macrophages were performed using FACS CantoII (BD Biosciences) and FlowJo 7.2.2. software (Tomy Digital Biology, Tokyo, Japan).

### Statistical Analysis

Data are expressed as means ± standard error of means (SEMs) and a P value of <0.05 was considered significant for all experiments. Statistical analysis was performed using Student’s t-test or ANOVA followed by Scheffe test, where appropriate. The statistical analysis in the survival curve was performed with the log-rank test. All of the experiments in this article were repeated more than 3 times.

## Additional Information

**Accession codes:** Microarray data have been deposited in the NCBI Gene Expression Omnibus under accession code GSE78153.

**How to cite this article**: Hachiya, R. *et al*. The H3K9 methyltransferase Setdb1 regulates TLR4-mediated inflammatory responses in macrophages. *Sci. Rep.* **6**, 28845; doi: 10.1038/srep28845 (2016).

## Supplementary Material

Supplementary Information
