# Supplementary material for: The H3K9 methyltransferase Setdb1 regulates TLR4-mediated inflammatory responses in macrophages
Source: Sci Rep. 2016 Jun 28;6:28845. doi: 10.1038/srep28845 (PMC4924096; doi:10.1038/srep28845)

Supplementary Information

The H3K9 methyltransferase Setdb1 regulates TLR4-mediated inflammatory responses in macrophages

Rumi Hachiya[1], Takuya Shiihashi[1,4], Ibuki Shirakawa[2], Yorihiro Iwasaki[1], Yoshihiro Matsumura[6], Yumiko Oishi[3], Yukiteru Nakayama[5], Yoshihiro Miyamoto[7], Ichiro Manabe[8], Kozue Ochi[9], Miyako Tanaka[9], Nobuhito Goda[4], Juro Sakai[6], Takayoshi Suganami[9,10*], and Yoshihiro Ogawa[1,11*]

[1]Department of Molecular Endocrinology and Metabolism, [2]Department of Organ Network and Metabolism, Graduate School of Medical and Dental Sciences, [3]Department of Cellular and Molecular Medicine, Medical Research Institute, Tokyo Medical and Dental University, Tokyo, Japan. [4]Department of Life Science and Medical Bio-Science, School of Advanced Science and Engineering, Waseda University, Tokyo, Japan. [5]Department of Cardiovascular Medicine, Graduate School of Medicine and Faculty of Medicine, [6]Division of Metabolic Medicine, Research Center for Advanced Science and Technology, University of Tokyo, Tokyo, Japan. [7]Department of Preventive Cardiology, National Cerebral and Cardiovascular Center, Osaka, Japan. [8]Department of Aging Research, Graduate School of Medicine, Chiba University, Chiba, Japan. [9]Department of Molecular Medicine and Metabolism, Research Institute of Environmental Medicine, Nagoya University, Nagoya, Japan. [10]Japan Science and Technology Agency, PRESTO, Tokyo, Japan. [11]Japan Agency for Medical Research and Development, AMED-CREST, Tokyo, Japan.

[*]Correspondence and requests for materials should be addressed to T.Su. (e-mail:suganami@riem.nagoya-u.ac.jp) or Y.Og. (e-mail: ogawa.mem@tmd.ac.jp)

Supplementary Table S1: Antibodies used in Western blotting and chromatin immunoprecipitation assay.

	Vendor	No.	Application
Setdb1	Dr. Sakai[16]	F0838	WB
Flag M2	Sigma-Aldrich	F3165	WB
NF-κB p65	Santa Cruz Biotechnology	sc-372	WB, ChIP
IκBα	Cell Signaling Technology	#9242	WB
JNK	Cell Signaling Technology	#9252	WB
p-JNK	Cell Signaling Technology	#9255	WB
p38	Cell Signaling Technology	#9212	WB
p-p38	Cell Signaling Technology	#9211	WB
ERK	Cell Signaling Technology	#9102	WB
p-ERK	Cell Signaling Technology	#9101	WB
Lamin A/C	Santa Cruz Biotechnology	sc-20681	WB
HDAC1	Santa Cruz Biotechnology	sc-6298	WB
H3K9me3	Abcam	#8898	ChIP

Supplementary Table S2: Antibodies used in flow cytometric analyses.

	Vendor	Clone	Conjugated
CD11b	BioLegend	M1/70	FITC
F4/80	BioLegend	BM8	APC/Cy7
CD11c	BD Pharmingen	HL3	PE
CD206	BioLegend	C068C2	APC
CD3ε	BioLegend	145-2C11	PE
CD45R (B220)	BioLegend	RA3-6B2	APC
Ly6G	BioLegend	1A8	APC/Cy7

Supplementary Table S3: PCR primers for mRNA expression.

Setdb1	Fw	AAGAGTGTCTGCCCACAGGG
	Rv	CATGTTTGGGTCACAATTGCA
IL6	Fw	ACAACCACGGCCTTCCCTACTT
	Rv	CACGATTTCCCAGAGAACATGTG
IL12b	Fw	ACATCTACCGAAGTCCAATGCA
	Rv	GGAATTGTAATAGCGATCCTGAGC
MyD88	Fw	CACGGTGGTGGTTGTTTCTGAC
	Rv	TGGAGACAGGCTGAGTGCAA
TLR2	Fw	AGAGAGTAGTTCGGCCTTTCC
	Rv	GTGCATCCATCATTGGGACAG
TLR4	Fw	AGAAATTCCTGCAGTGGGTCA
	Rv	TCTCTACAGGTGTTGCACATGTCA
TRAF2	Fw	AGAGAGTAGTTCGGCCTTTCC
	Rv	GTGCATCCATCATTGGGACAG
TRAF6	Fw	AAAGCGAGAGATTCTTTCCCTG
	Rv	ACTGGGGACAATTCACTAGAGC
NF-κB p65	Fw	TGTGGAGATCATCGAACAGCCG
	Rv	TTCCTGGTCCTGTGTAGCCATTGAT
NF-κB1 p50	Fw	CACTGCTCAGGTCCACTGTC
	Rv	CTGTCACTATCCCGGAGTTCA
NF-κB2 p52	Fw	GGCCGGAAGACCTATCCTACT
	Rv	CTACAGACACAGCGCACACT
c-Rel	Fw	GCCGTGTGAACAAGAACTGTGGAA
	Rv	TTGTGAAAAAACACCTCTGGCTTCC
CD14	Fw	CGAACAAGCCCGTGGAACCT
	Rv	CAAGCACACGCTCCATGGTC
LBP	Fw	TCGCCATCTCTGACTCTTCC
	Rv	GGAGGTCCACTGAAATGGTG
MD-2	Fw	CGCTGCTTTCTCCCATATTGA
	Rv	CCTCAGTCTTATGCAGGGTTCA
TIRAP	Fw	CCTCCTCCACTCCGTCCAA
	Rv	CTTTCCTGGGAGATCGGCAT

TRIF	Fw	TCCCTTCTCTCTCCTCCTCCTC
	Rv	CAGCACAGACCTGGCTGTAGG
TRAM	Fw	GCAGTACCACTTCCCAGCTAA
	Rv	ACTCATTTGACACTGGGCTCT

Supplementary Table S4: PCR primers for ChIP assay.

IL6	Fw	CCTGCGTTTAAATAACATCAGCTTTAGCTT
	Rv	GCACAATGTGACGTCGTTTAGCATCGAA
GAPDH (negative control for H3K9me ChIP)	Fw	ATCCTGTAGGCCAGGTGATG
	Rv	AGGCTCAAGGGCTTTTAAGG
GAPDH (negative control for NF-κB p65 ChIP)	Fw	AATGTGTCCGTCGTGGATCT
	Rv	CATCGAAGGTGGAAGAGTGG

Supplementary Table S5: The 42 genes upregulated by lipid A treatment in KO macrophages.

Gene symbol	WT (Lipid A vs. Veh) Fold change	KO vs. WT (Lipid A) Fold change
Il1b	12.5	42.2
Mmp13	2.3	15.9
Ccl2	14.9	14.1
Il1a	6.1	12.0
Plagl1	3.7	9.8
Ccl7	4.6	7.9
Il12b	14.1	7.8
Il6	1910.6	6.8
Ccnd2	4.7	6.0
Slc7a2	7.4	5.7
Cxcl1	23.5	5.6
Ppfia3	4.1	5.4
Socs3	4.2	5.3
LOC433762	5.6	5.2
Ptx3	5.8	5.0
Ptgs2	53.3	5.0
A130040M12Rik	2.2	4.9
Hbegf	2.5	4.8
Inhba	3.9	4.7
Flnb	2.1	4.7
Ccl4	20.0	4.4
Cd40	23.1	4.0
Aqp9	3.6	3.9
Ear-ps9	4.1	3.9
Rhox2g	4.1	3.9
Cd83	3.3	3.8
Olr1	6.6	3.7
Cav1	4.8	3.6
Mki67	10.8	3.6
Niacr1	19.8	3.5
Bcl2l11	2.5	3.5
Zc3h12a	2.1	3.4

Ier3	3.4	3.3
Slfn4	10.7	3.3
Flrt3	3.1	3.3
Stat5a	2.6	3.2
Ccrn4l	4.0	3.2
Ccl3	9.4	3.2
Sdc4	4.7	3.1
Tslp	14.1	3.1
Tpbg	33.5	3.0
Sh3bp5	7.3	3.0

Supplementary Table S6: Gene ontology analysis of the 42 genes.
The top 10 significantly enriched categories are shown.

Pathway name	Entities p value	Submitted entities found	# Entities found	# Entities total
Chemokine receptors bind chemokines	1.5E-08	Cxcl1;Ccl2;Ccl3;Ccl7;Ccl4	7	55
Peptide ligand-binding receptors	6.1E-05	Cxcl1;Ccl2;Ccl3;Ccl7;Ccl4	7	194
Transport of glycerol from adipocytes to the liver by Aquaporins	1.2E-04	Aqp9	2	3
Signaling by Interleukins	4.5E-04	Socs3;Il1a;Il1b;Stat5a;Il1a;Il6;Il6	5	124
Interleukin-1 processing	6.3E-04	Il1a;Il1b;Il1a	2	7
Class A/1 (Rhodopsin-like receptors)	7.4E-04	Cxcl1;Ccl2;Ccl3;Niacr1;Ccl7;Ccl4	8	384
Senescence-Associated Secretory Phenotype (SASP)	1.0E-03	Il1a;Il1a;Il6;Il6	4	85
Interleukin-6 signaling	3.2E-03	Socs3;Il6;Il6	2	16
Passive transport by Aquaporins	4.9E-03	Aqp9	2	20
Signaling by Leptin	7.0E-03	Socs3;Stat5a	2	24

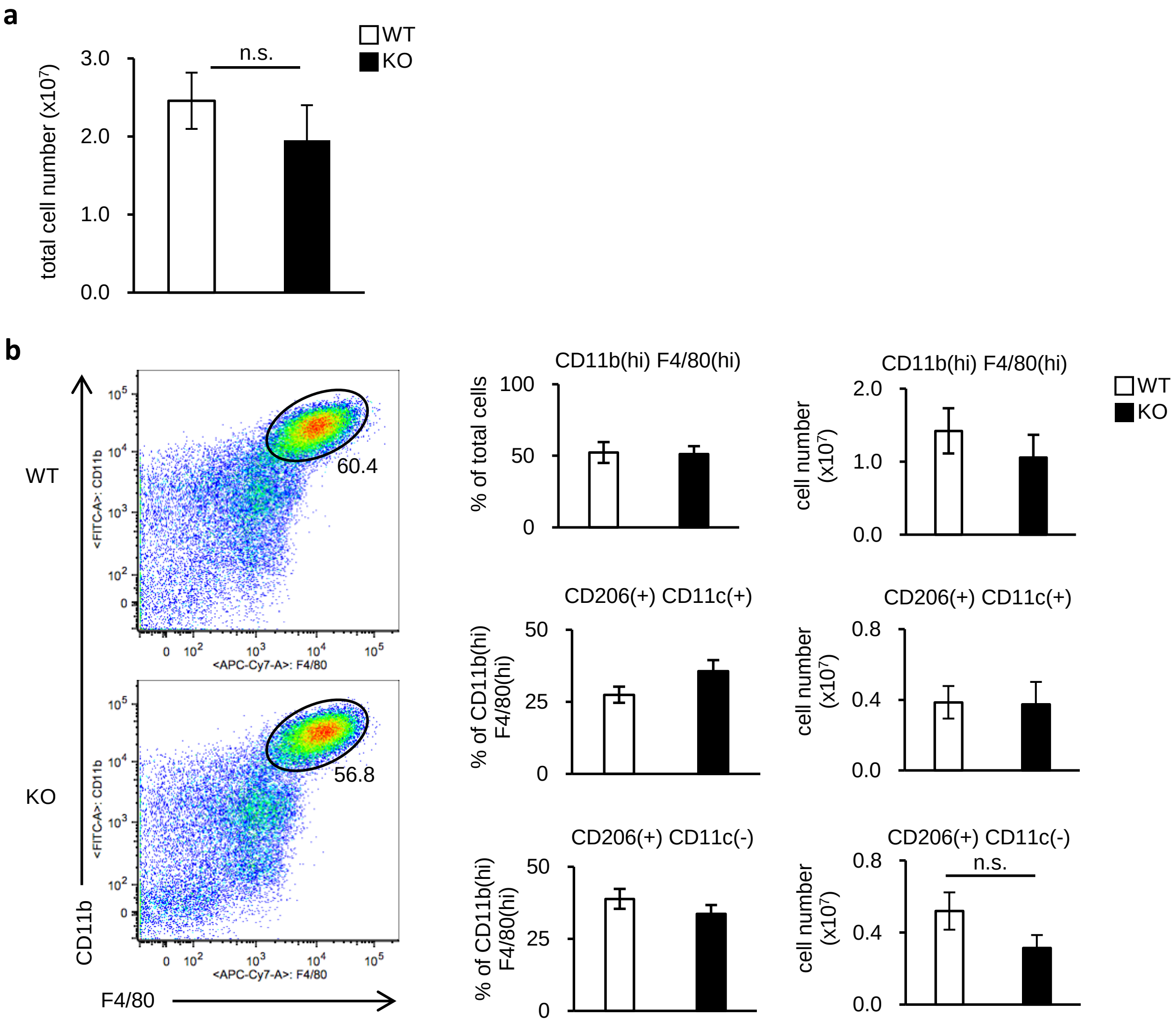

Supplementary Figure S1. Characterization of thioglycollate-elicited exudate cells from Setdb1-deficient (KO) and wildtype (WT) mice.

(a) The total exudate cell number. (b) Representative FACS plots to detect peritoneal macrophages using anti-CD11b and anti-F4/80 antibodies. Peritoneal macrophages were defined as CD11b^{hi} F4/80^{hi} cells, and the numbers in the plots indicate the percentage in the total exudate cells. The polarized macrophages were analyzed using anti-CD206 and anti-CD11c antibodies (n.s.: not significant, n = 7).

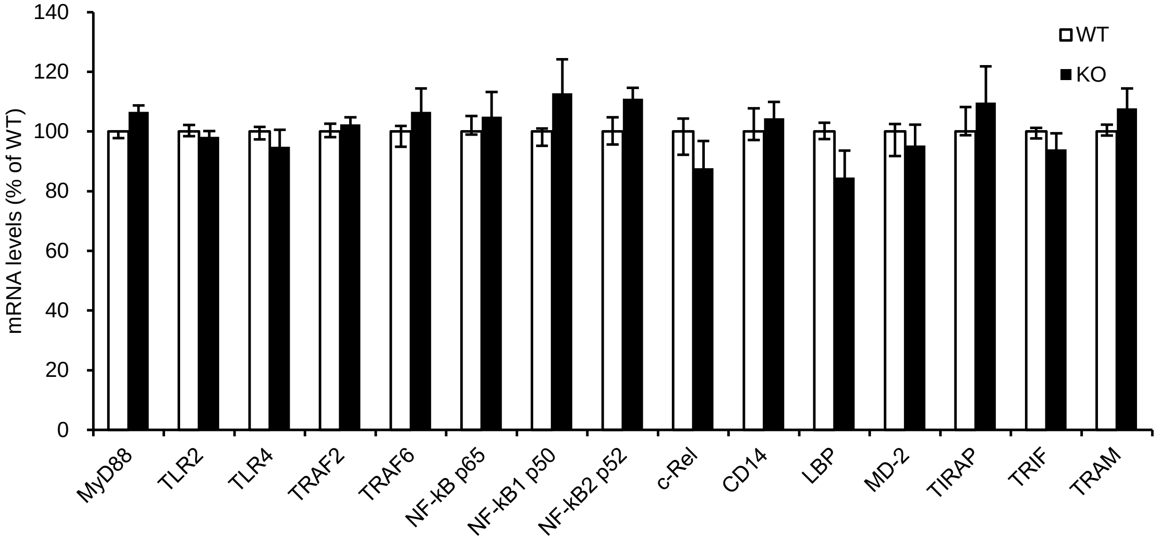

Supplementary Figure S2. Setdb1 is dispensable for expression of genes related to the TLR4 signaling pathways.

KO and WT peritoneal macrophages were treated with lipid A (10 ng/ml) for 4 hours, and mRNA expression levels were analyzed by quantitative real-time PCR (n = 3).

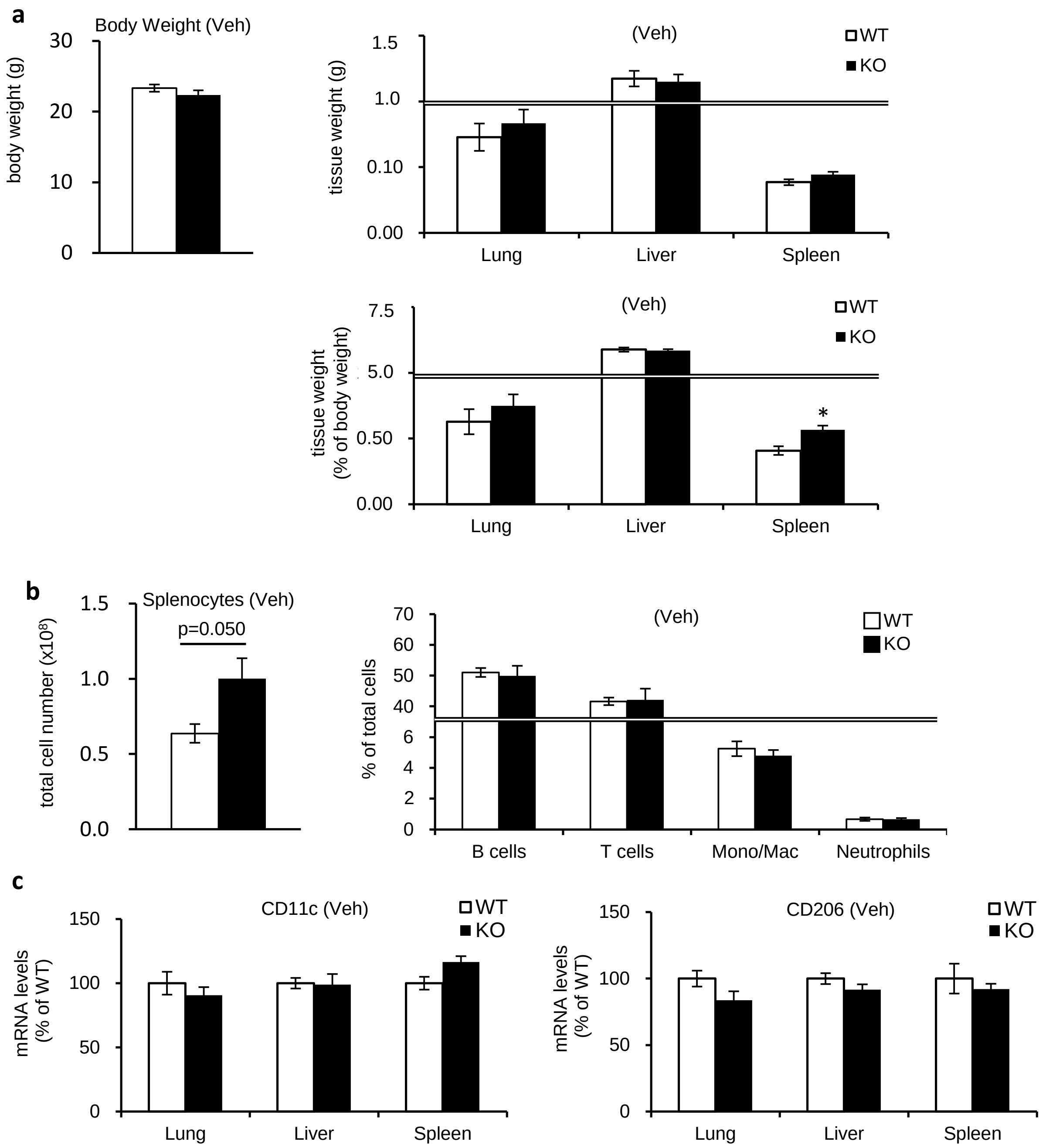

Supplementary Figure S3. Baseline characteristics of vehicle-treated Setdb1-deficient mice.
(a) Body weight and tissue weights (*P < 0.05; WT, n = 5; KO, n = 10). (b) The total cell number and population of splenocytes were analyzed with flow cytometry. B lymphocytes, $B220^+$; T lymphocytes, $CD3e^+$; monocytes/macrophages, $CD11b^+Ly6G^-$; neutrophils, $CD11b^+Ly6G^+$. (c) mRNA expression of CD11c and CD206 in lung, liver, and spleen.

Supplementary Figure S4. Full images of Western blots

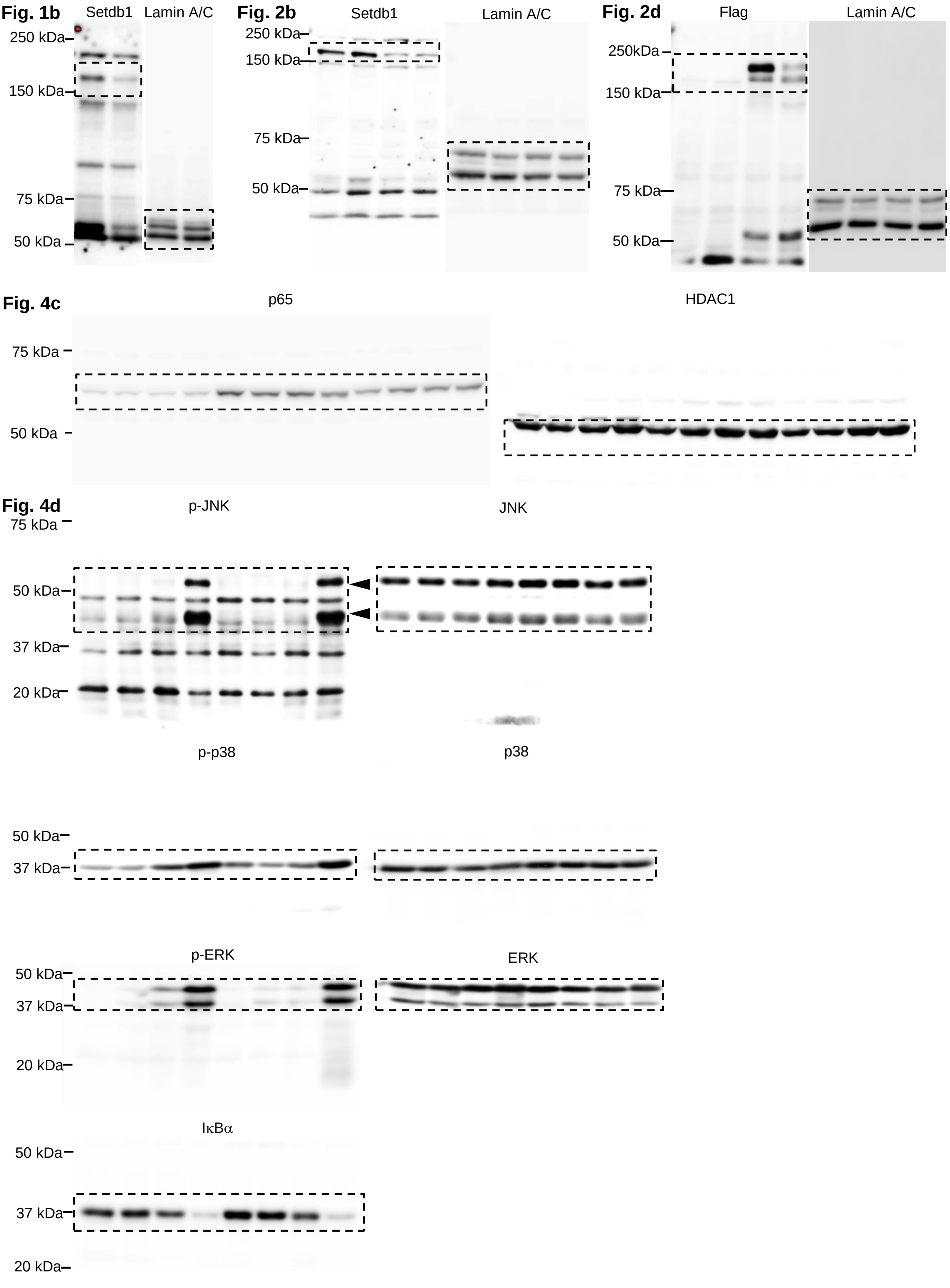

Supplementary Figure S4. Full images of Western blots (continued)

Fig. 4e

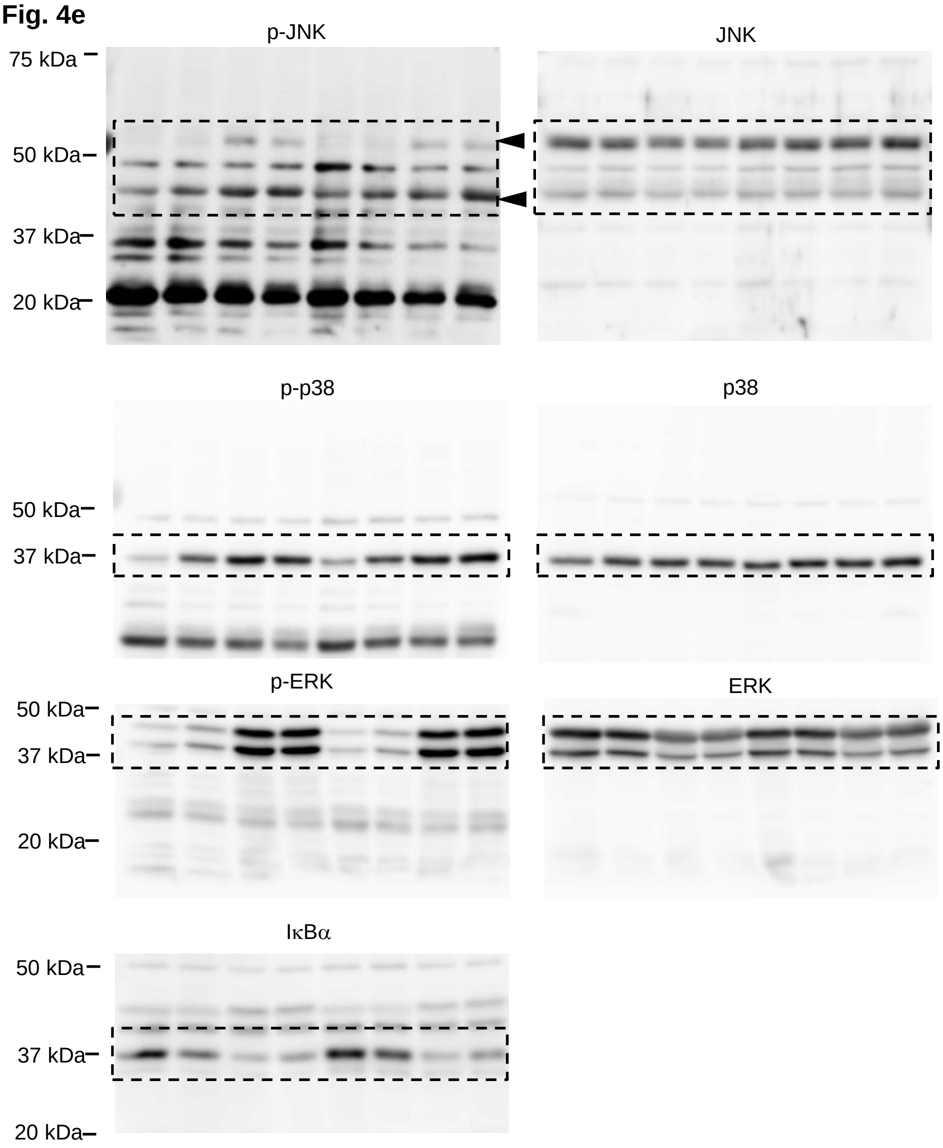

Supplement: Supplementary Information [file srep28845-s1.pdf]
